# Factors associated with frailty in a community-dwelling population of older adults

**DOI:** 10.11606/S1518-8787.2017051007098

**Published:** 2017-11-13

**Authors:** Danielle Teles da Cruz, Marcel de Toledo Vieira, Ronaldo Rocha Bastos, Isabel Cristina Gonçalves Leite

**Affiliations:** I Universidade Federal de Juiz de Fora. Faculdade de Medicina. Departamento de Saúde Coletiva. Juiz de Fora, MG, Brasil; II Universidade Federal de Juiz de Fora. Departamento de Estatística. Juiz de Fora, MG, Brasil

**Keywords:** Aged, Frail Elderly, Aging, Health Vulnerability, Risk Factors, Health Surveys, Idoso, Idoso Frágil, Envelhecimento, Vulnerabilidade em Saúde, Fatores de Risco, Inquéritos Epidemiológicos

## Abstract

**OBJECTIVE:**

To analyze if demographic and socioeconomic factors and factors related to health and health services are associated with frailty in community-dwelling older adults.

**METHODS:**

This is a cross-sectional study with 339 older adults (60 years old or more) living in Juiz de Fora, State of Minas Gerais, Brazil, in 2015. A household survey was carried out and frailty was evaluated using the Edmonton Frail Scale. For the analysis of the factors associated with outcome, a theoretical model of determination was constructed with three hierarchical blocks: block 1 with demographic and socioeconomic characteristics, block 2 with the health of the older adult (divided into three sub-levels: 2.1 self-reported health variables, 2.2 self-perceived health variables, and 2.3 geriatric syndromes), and block 3 with characteristics related to health services. The variables were adjusted in relation to each other within each block; those with significance level ≤ 0.20 were included in the Poisson regression model and adjusted to a higher level, considering a level of significance of 5%.

**RESULTS:**

The prevalence of frailty among older adults was 35.7% (95%CI 30.7–40.9). Of the total, 42.2% did not present frailty; 22.1% were apparently vulnerable. Among the frail ones, 52.9% presented mild frailty, 32.2% moderate frailty, and 14.9% severe frailty. Frailty was associated with difficulty walking, need for an auxiliary device to walk, presence of caregiver, depressive disorders, and functional dependence to perform instrumental activities of daily living.

**CONCLUSIONS:**

Frailty is frequent among the older population and it is associated with health variables of the three sub-levels that make up block 2 of the theoretical hierarchical model of determination: self-reported health variables, self-perceived health variables, and geriatric syndromes.

## RESUMO

**OBJETIVO:**

Analisar se fatores demográficos e socioeconômicos e fatores referentes à saúde e aos serviços de saúde estão associados à fragilidade em idosos comunitários.

**MÉTODOS:**

Estudo transversal com 339 idosos (60 anos ou mais) residentes em Juiz de Fora, MG, em 2015. Foi realizado inquérito domiciliar e a fragilidade foi avaliada por meio da Escala de Fragilidade de Edmonton. Para a análise dos fatores associados ao desfecho, foi construído um modelo teórico de determinação com três blocos hierarquizados: bloco 1, com características demográficas e socioeconômicas; bloco 2, saúde do idoso (dividido em três subníveis: 2.1 variáveis de saúde referidas; 2.2 variáveis de saúde autopercebidas e 2.3 síndromes geriátricas); e o bloco 3, com características referentes aos serviços de saúde. As variáveis foram ajustadas entre si dentro de cada bloco; aquelas com nível de significância ≤ 0,20 foram incluídas no modelo de regressão de Poisson e ajustadas ao nível superior ao seu, considerando o nível de 5% de significância.

**RESULTADOS:**

A prevalência de fragilidade entre os idosos foi de 35,7% (IC95% 30,7–40,9). 42,2% não apresentaram fragilidade; 22,1% eram aparentemente vulneráveis. Entre os frágeis, 52,9% apresentaram fragilidade leve, 32,2% fragilidade moderada e 14,9% fragilidade grave. Fragilidade associou-se com dificuldade para andar, necessidade de dispositivo auxiliar para locomoção, presença de cuidador, transtornos depressivos e dependência funcional para realização das atividades instrumentais de vida diária.

**CONCLUSÕES:**

A fragilidade é frequente entre a população idosa e está associada a variáveis de saúde dos 3 subníveis que compõe o bloco 2 do modelo teórico hierárquico de determinação: variáveis de saúde referidas; variáveis de saúde autopercebidas e síndromes geriátricas.

## INTRODUCTION

Population aging is one of the most striking phenomena in contemporary societies and it presents important developments and impacts for society and health systems. According to the World Health Organization^a^, the Brazilian older population will increase from the current 12.5% to approximately 30% by 2050. The magnitude of this process in the country makes it necessary to understand the demands related to the health of older adults^a^.

The situation becomes even more challenging for countries such as Brazil, where a number of particularities are present that potentiate the negative impacts of population aging on the social security system and, consequently, on the health of older adults. Among the main aspects to be observed in this context, we can mention poverty, low education level, social inequality, gender issues, abuse, lack of formal social support, low pension values, high prevalence of multiple chronic diseases, lack of leisure, misinformation, prejudice and disrespect, and the incongruity of the health system in the face of aging populations^13^ ^,^ ^a^.

In this context, frailty should be understood as a public health priority, since it is highly prevalent, negatively affects the quality of life of older adults and their families, and demands high social and economic costs. In addition, it is an important predictor of falls, functional disability, hospitalizations, comorbidities, complications of existing diseases, institutionalization, and mortality^3^ ^,^ ^4^ ^,^ ^11^ ^,^ ^17^ ^,^ ^22^.

Frailty can be understood as a multifactorial, multifaceted, dynamic, syndromic condition, resulting from the existing arrangement between the biological, social, psychological, and environmental aspects that interact with each other in the course of human life and the relationships that are processed within it. Thus, health-related vulnerabilities must extrapolate the physical dimension and cannot be disassociated from domains such as cognition, humor, and social support^3^ ^,^ ^4^ ^,^ ^22^.

Population studies that use this concept and that evaluate frailty and associated factors in community-dwelling older adults are scarce in Brazil. The objective of this study was to analyze the factors associated with frailty in community-dwelling older adults.

## METHODS

This is a cross-sectional, population-based study carried out with a household survey between October 2014 and March 2015, with a sample of 339 older adults aged 60 years or more living in the Northern Zone of the city of Juiz de Fora, State of Minas Gerais, Brazil. This study is part of a larger research project, and it corresponds to a cross-sectional profile of the second phase of a cohort, which begun in 2010.

The 2010 study consisted of 420 older adults and the participants were selected by stratified and conglomerate random sampling in multiple stages. The primary units were the census tracts. For the random selection, the tracts were grouped into strata defined according to the different types of health care to which the population of the tract was assigned, subdivided into primary care (Family Health Strategy [FHS] or traditional), secondary care, or uncovered area. We selected these tracts with probabilities proportional to their size (resident population, according to the 2000 Demographic Census), independently, in each stratum^5^.

For the 2014 survey, we estimated the calculation of the sample size from data from the previous work and from the results of the 2010 IBGE census related to the population of the delimited area, at the level of census tract breakdown. There were changes in the population and in the distribution of these tracts, which required the resizing of the representative probabilistic sample based on stratification and conglomeration. In order to neutralize the loss of panel members, who ceased to be part of the population surveyed over the years, we used the over sample method, allowing the initial sampling to be respected, as long as the initial population is known and statistical treatment and weight assignment are different between the groups that make up each situation of the panel member lost (cases of death, change of address without being able to identify the new address, long-term travel, long-term hospitalization, and entry into long-term institution)^21^. We selected age, sex and education level as the variables to mark the entry of new subjects. Thus, 248 older adults of the 2010 study and 175 new older adults (amounting to 423 older adults) made up the sample for the 2014 study.

The Mini-Mental State Examination (MMSE) was used as a screening tool for cognitive decline, linked to the process of both senility and senescence, which determined the need or not of another respondent. In the case of another respondent, the issues that require the self-perception of the older adult were not addressed. Researchers state that the education level influences the performance of the MMSE and the adoption of stratified cutoff points decreases diagnostic failures, since the education of the Brazilian population is quite diversified and the education level of most older adults is low^2^ ^,^ ^19^. However, to date, there is no consensus on the cutoff points to be used in Brazil^20^.

From this perspective, we used the cutoff point used by the State Health Department of Minas Gerais, which uses this instrument for the evaluation of older adults. The minimum expected score for older adults with four years or more of education is 25 points, and the expected score for older adults with less than four years of education is 18 points. Lower scores indicate cognitive impairment^b^. Individuals who presented behavior in the MMSE suggestive of cognitive decline and who were not accompanied by family members or caregivers were excluded (n = 23).

The evaluation of frailty was performed using the Edmonton Frail Scale (EFS)^22^, adapted and validated for the Brazilian population^9^. The scale has eleven items that evaluate nine domains: cognition, general health status, functional independence, social support, drug use, nutrition, humor, continence, and functional performance. The total score can vary from zero to seventeen points: no frailty (0 to 4), apparently vulnerable (5 and 6), mild frailty (7 and 8), moderate frailty (9 and 10), and severe frailty (11 points or more). The outcome variable was dichotomized according to the frailty scores: frail and non-frail with cutoff point ≥ 7 points.

The first item of the EFS, the clock test, determines the respondent of this instrument: if the older adult fails it, the caregiver is responsible for responding the instrument. Older adults who were reproved with significant errors and who did not have a caregiver (n = 61) were excluded from the study on frailty^9^ ^,^ ^23^.

The questionnaire used to identify the sociodemographic profile and health issues was standardized and pretested. The Patient Health Questionnaire-4 (PHQ-4) was also used to track anxiety and depression disorders, the Falls Efficacy Scale - International - Brazil was used to evaluate fear of falling, and the Lawton and Brody scale^16^ was used to evaluate the functional capacity to perform instrumental activities of daily living (IADL).

The Lawton and Brody scale, although not having satisfactory standards of adaptation and validation for Brazil, is widely used in national research studies^1^ ^,^ ^10^ ^,^ ^24^ and is mentioned as a tool for the functional evaluation of older adults in primary care, since 2006, by the Ministry of Health^c^, and also by the Health Department of the State of Minas Gerais^b^.

Absolute and relative frequencies were described, as well as the prevalence of the outcome. The chi-square test was used to analyze the association of the dependent variable with the independent variables in the bivariate analysis. Using Poisson regression, we analyzed the independent variables associated with outcome, controlling possible confounding factors (adjusted PR) in the multiple analysis. Statistical significance was analyzed using the Wald tests for heterogeneity and linear trend.

For the analysis of the factors associated with frailty, we constructed a theoretical determination model with three hierarchical blocks of variables (Figure), adjusted for each other within each block. Variables with significance level ≤ 0.20 were included in the Poisson regression model and adjusted to a higher level.

**Figure:**
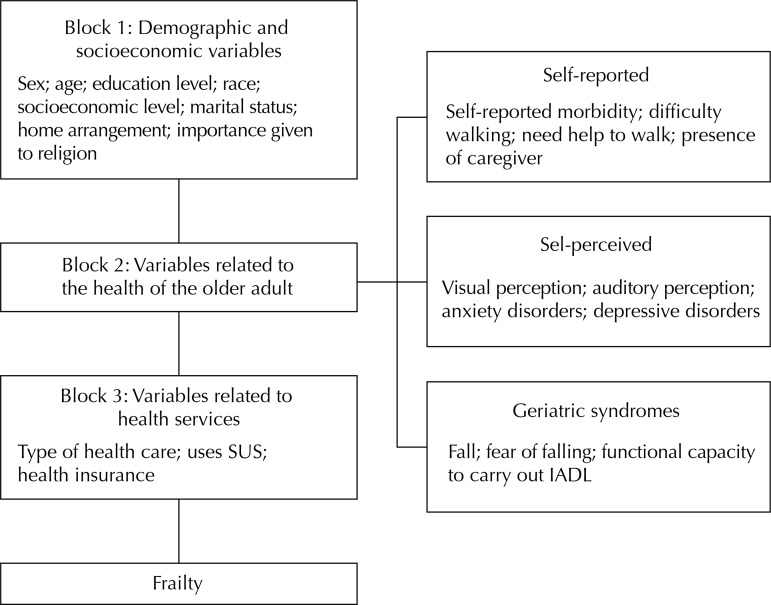
Theoretical model of investigation of the effects of the independent variables on frailty in hierarchical blocks.

SUS: Brazilian Unified Health System; IADL: instrumental activities of daily living

The independent variables were grouped into three blocks: Block 1 with demographic and socioeconomic characteristics, Block 2 with the health of the older adult (divided into three sub-levels: 2.1 self-reported health variables, 2.2 self-perceived health variables, and 2.3 geriatric syndromes), and Block 3 with characteristics related to health services.

We used the software Statistical Package for Social Sciences (SPSS) version 15.0 in its module of complex samples, which considers the characteristics of the sample plan and significance level of 5%, and Stata 9.2, also considering the sample plan (module of survey data analysis).

We followed the Regulatory Standards and Directives of Research Involving Human Beings, according to Resolution 466 of the National Health Council. The Ethics Committee of the Universidade Federal de Juiz de Fora has approved the study (Process 771/916). The informed consent was read and signed by all participants.

## RESULTS

The sample consisted of 61.1% of women. Average age was 74.3 years (SD = 8.24) and average education level was 4.2 years of study (SD = 3.47). Among the participants, 47.8% declared themselves white, 59% belonged to socioeconomic level C, 61.7% were married or had a common-law marriage, and 93.8% lived with someone. Catholics were 76.7% of the interviewees and 96.9% classified religion as important for their lives. Morbidity was reported by 89.4% of the older adults, walking difficulty was reported by 43.1% of the individuals, and 81.1% said they did not need help to walk. The need for the continued use of at least one drug was reported by 92% of the sample. A large proportion (44.5%) had a caregiver (96.0% of them had family members or friends as caregivers). Poor or regular health perception was reported by 41.6% of the older adults, 53.7% reported regular or poor health in relation to vision, and 27.1% in relation to hearing (Table 1).

**Table 1:** Characteristics of the sample according to independent variables. Juiz de Fora, State of Minas Gerais, Brazil, 2015.

Variable	Presence of frailty		Absence of frailty	
	
	n	%	n	%
Block 1 – Demographic and socioeconomic variable				

Sex				
Male	38	32.5	94	42.3
Female	79	67.5	128	57.7
Age (years)				
60–70	26	22.2	103	46.4
71–80	41	35.1	80	36.0
More than 80	50	42.7	39	17.6
Education level				
Illiterate	21	17.9	21	9.5
1–4 years	80	68.4	129	58.1
5–10 years	14	12.0	48	21.6
11 years or more	2	1.7	24	10.8
Race				
White	56	47.9	106	47.7
Black	20	17.1	30	13.5
Brown	35	29.9	70	31.5
Yellow/Indigenous	6	5.1	16	7.3
Socioeconomic level				
A or B	31	26.5	77	34.7
C	71	60.7	129	58.1
D or E	15	12.8	16	7.2
Marital status				
Married or common-law marriage	55	47.1	154	69.4
Widow	52	44.4	49	22.0
Divorced or separated	8	6.8	12	5.4
Single	2	1.7	7	3.2
Home arrangement				
Lives alone	8	6.8	13	5.9
Lives with someone	109	93.2	209	94.1
Religion				
None	1	0.9	7	3.2
Catholic	91	77.8	169	76.1
Protestant or Evangelical	23	19.6	38	17.1
Other	2	1.7	8	3.6
Importance given to religion*				
Important	61	98.4	186	96.4
More or less important	0	0	5	2,6
Not important	1	1.6	2	1.0

Block 2.1 – Variable related to the health of the older adult: self-reported				

Self-reported morbidity				
No	6	5.1	30	13.5
Yes	111	94.9	192	86.5
Difficulty walking				
No	28	23.9	165	74.3
Yes	89	76.1	57	25.7
Need help to walk				
No	62	53.0	213	95.9
Human help	23	19.7	4	1.8
Auxiliary device	32	27.3	5	5.3
Medication of continuous use				
None	1	0.9	26	11.7
1–4	36	30.8	133	59.9
More than 4	80	68.3	63	28.4
Presence of caregiver				
No	28	23.9	160	72.0
Family/Friend	84	71.8	61	27.5
Hired	5	4.3	1	0.5

Block 2.2 – Variable related to the health of the older adult: self-perceived				

Self-perception of health*				
Excellent/Very good/Good	13	21.0	136	70.5
Regular/Poor	49	79.0	57	29.5
Visual perception				
Excellent/Very good/Good	15	24.2	103	53.4
Regular/Poor	47	75.8	90	46.6
Auditory perception*				
Excellent/Very good/Good	40	64.5	146	75.6
Regular/Poor	22	35.5	47	24.4
Anxiety disorders*				
No	34	54.8	157	81.3
Yes	28	45.2	36	18.7
Depressive disorders*				
No	36	58.1	170	88.1
Yes	26	41.9	23	11.9

Block 2.3 – Variable related to the health of the older adult: geriatric syndromes				

Fall				
No	62	53.0	156	70.3
Yes	55	47.0	66	29.7
Fear of falling*				
No	1	1.6	10	5.2
Yes	61	98.4	183	94.8
Functional capacity to carry out IADL				
Independent	65	55.6	217	97.7
Dependent	52	44.4	5	2.3

Block 3 – Variable related to health services				

Type of health care				
BHU with FHS	85	72.6	163	73.4
Traditional BHU	32	27.4	59	26.6
Uses SUS				
Yes	114	97.4	212	95.5
No	3	2,6	10	4.5
Health insurance				
Yes	67	57.3	134	60.4
No	50	42.7	88	39.6

IADL: instrumental activities of daily living; BHU: basic health unit; FHS: Family Health Strategy; SUS: Brazilian Unified Health System

* Variables investigated only when the respondent was the older adult.

Depression and anxiety disorders were observed in 19.2% and 25.1% of the older adults, respectively. Regarding geriatric syndromes, 35.7% reported a fall in the last year, 95.7% were afraid of falling, and 16.8% presented functional dependence. Most of the older adults lived in areas where the main type of health care was the FHS, reported using the SUS for appointments, hospitalizations, exams, vaccination, or participation in educational groups, and reported having health insurance (Table 1).

The prevalence of frailty was 35.7% (95%CI 30.7–40.9). Of the total, 42.2% did not present frailty, 22.1% were apparently vulnerable, and, among the frail ones, 18.9% presented mild frailty, 11.5% moderate frailty, and 5.3% severe frailty.

Frailty was associated with females, age increase, socioeconomic levels D or E, low education level, widowhood, difficulty walking, and the need for an auxiliary device to walk. Frailty was more frequent among those who had a caregiver (p < 0.001), poor or regular perception of vision (p < 0.001), depression (p < 0.001) and anxiety disorders (p < 0.001), those who reported falls (p = 0.002), and those with functional dependency (p < 0.001) (Table 2).

**Table 2:** Crude and adjusted prevalence ratios between the hierarchical blocks for the occurrence of frailty. Juiz de Fora, State of Minas Gerais, Brazil, 2015.

Variable	%	Crude PR (95%CI)	p	Adjusted PR (95%CI)	p
Block 1 – Demographic and socioeconomic variable					

Sex^a^			0.098		0.664
Male	28.8	1		1	
Female	38.2	1.53 (0.96–2.44)		1.13 (0.64–1.99)	
Age (years)^b^			< 0.001		
60–70	20.2	1		1	0.001
71–80	33.9	2.03 (1.15–3.60)		2.01 (1.15–3.82)	
More than 80	56.2	5.08 (2.79–9.26)		3.71 (1.87–7.37)	
Education level^b^			< 0.001		
11 years or more	7.7	1		1	0.028
5–10 years	22.6	3.50 (0.74–16.67)		1.02 (0.49–2.11)	
1–4 years	38.3	7.44 (1.71–32.34)		2.16 (0.86–5.42)	
Illiterate	50.0	12.00 (2.51–57.35)		6.69 (1.21–37.11)	
Race^b^			0.684		
Black	40.0	1		-	-
White	34.6	0.79 (0.41–1.52)		-	-
Brown	33.3	0.75 (0.37–1.51)		-	-
Yellow/Indigenous	27.3	0.56 (0.19–1.68)		-	-
Socioeconomic level (ABEP)^b^			0.044		
A or B	28.7	1		1	0.955
C	35.5	1.37 (0.82–2.27)		1.10 (0.48–2.51)	
D or E	48.4	2.33 (1.03–5.28)		1.15 (0.47–2.82)	
Marital status^b^			0.012		
Married or common-law marriage	26.3	1		1	0.097
Widow	51.5	2.97 (1.81–4.89)		0.95 (0.16–5.67)	
Divorced or separated	40.0	1.87 (0.73–4.81)		0.62 (0.09–4.38)	
Single	22.2	0.80 (0.16–3.97)		1.76 (0.32–9.77)	
Home arrangement^a^			0.905		
Lives with someone	34.1	1		-	-
Lives alone	38.1	1.18 (0.48–2.93)		-	-
Importance given to religion^a^			0.684		
Important	24.7	1		-	-
Not or little important	12.5	0.44 (0.05–3.61)		-	-

Block 2.1 – Variable related to the health of the older adult: self-reported					

Self-reported morbidity^a^			0.028		0.705
No	16.7	1		1	
Yes	36.6	2.89 (1.17–7.16)		1.24 (0.41–3.74)	
Difficulty walking^a^			< 0.001		< 0.001
No	14.5	1		1	
Yes	61.0	9.20 (5.47–15.49)		4.45 (2.42–8.19)	
Need help to walk^b^			< 0.001		
No	22.5	1		1	< 0.001
Human help	85.2	19.75 (6.58–59.27)		1.04 (0.23–4.73)	
Auxiliary device	86.5	21.99 (8.22–58.82)		7.13 (2.46–20.65)	
Presence of caregiver^a^			< 0.001		< 0.001
No	14.9	1		1	
Yes	58.9	8.20 (4.90–13.74)		5.08 (2.80–9.20)	

Block 2.2 – Variable related to the health of the older adult: self-perceived					

Visual perception^a^			< 0.001		0.001
Excellent/Very good/Good	12.7	1		1	
Regular/Poor	34.3	3.59 (1.88–6.84)		3.29 (1.66–6.49)	
Auditory perception^a^			0.121		
Excellent/Very good/Good	21.5	1		-	-
Regular/Poor	31.9	1.71 (0.92–3.16)		-	-
Anxiety disorders^a^			< 0.001		0.052
No	17.8	1		1	
Yes	43.8	3.59 (1.94–6.66)		2.04 (1.00–4.17)	
Depressive disorders^a^			< 0.001		0.001
No	17.5	1		1	
Yes	53.1	5.34 (2.74–10.39)		3.72 (1.74–7.92)	

Block 2.3 – Variable related to the health of the older adult: geriatric syndromes					

Fall^a^			0.002		0.036
No	28.4	1		1	
Yes	45.5	2.10 (1.32–3.33)		1.79 (1.04–3.10)	
Fear of falling^a^			0.305		
No	9.1	1		-	-
es	25.0	3.33 (0.42–26.57)		-	-
Functional capacity to carry out IADL^a^			< 0.001		< 0.001
Independent	23.0	1		1	
Dependent	91.2	34.72 (13.31–90.55)		32.97 (12.60–86.30)	

Block 3 – Variable related to health services					

Type of health care^a^			0.981		
BHU with FHS	34.3	1		-	-
Traditional BHU	35.2	1.53 (0.96–2.44)		-	-
Uses SUS^a^			0.554		
Yes	35.0	1		-	-
No	23.1	0.56 (0.15–2.07)		-	-
Health insurance^a^			0.663		
Yes	33.3	1		-	-
No	36.2	1.14 (0.72–1.80)		-	-

ABEP: *Associação Brasileira de Empresas de Pesquisa*; IADL: instrumental activities of daily living; BHU: basic health unit; FHS: Family Health Strategy; SUS: Brazilian Unified Health System

^a^ p-value for heterogeneity.

^b^ p-value for linear trend.

Five variables remained associated with frailty in the multiple regression model (Table 3). Among the variables of block 2.1, difficulty walking (adjusted PR = 4.27, 95%CI 1.74–10.52), need for an auxiliary device to walk (adjusted PR = 9.42, 95%CI 2.06–43.16), and presence of caregiver (adjusted PR = 3.34, 95%CI 1.42–7.85) were risk factors. Depressive disorder (adjusted PR = 3.47, 95%CI 1.27–9.50) in block 2.2 and functional dependence for IADL (adjusted PR = 5.84, 95%CI 1.00–34.87) in block 2.3 were risk factors. No variables of the most distal (block 1) and proximal (block 3) levels presented statistical significance after the adjusted analysis.

**Table 3:** Multiple regression analysis in hierarchical blocks for the occurrence of frailty among older adults. Juiz de Fora, State of Minas Gerais, Brazil, 2015.

Variable	%	Crude PR (95%CI)	p	Adjusted PR (95%CI)	p
Block 1 – Demographic and socioeconomic variable					

Age (years)^b^			< 0.001		
60–70	20.2	1		1	0.214
71–80	33.9	2.03 (1.15–3.60)		1.35 (0.49–3.72)	
More than 80	56.2	5.08 (2.79–9.26)		2.68 (0.85–8.43)	
Education level^b^			< 0.001		
11 years or more	7.7	1		1	0.756
5–10 years	22.6	3.50 (0.74–16.67)		0.57 (0.04–7.72)	
1–4 years	38.3	7.44 (1.71–32.34)		0.46 (0.04–5.34)	
Illiterate	50.0	12.00 (2.51–57.35)		0.82 (0.06–11.61)	

Block 2.1 – Variable related to the health of the older adult: self-reported					

Difficulty walking^a^			< 0.001		0.002
No	14.5	1		1	
Yes	61.0	9.20 (5.47–15.49)		4.27 (1.74–10.52)	
Need help to walk^b^			< 0.001		
No	22.5	1		1	0.011
Human help	85.2	19.75 (6.58–59.27)		3.69 (0.49–27.93)	
Auxiliary device	86.5	21.99 (8.22–58.82)		9.42 (2.06–43.16)	
Presence of caregiver^a^			< 0.001		0.006
No	14.9	1		1	
Yes	58.9	8.20 (4.90–13.74)		3.34 (1.42–7.85)	

Block 2.2 – Variable related to the health of the older adult: self-perceived					

Visual perception^a^			< 0.001		0.094
Excellent/Very good/Good	12.7	1		1	
Regular/Poor	34.3	3.59 (1.88–6.84)		2.17 (0.88–5.38)	
Anxiety disorders^a^			< 0.001		0.269
No	17.8	1		1	
Yes	43.8	3.59 (1.94–6.66)		1.75 (0.65–4.72)	
Depressive disorders^a^			< 0.001		0.015
No	17.5	1		1	
Yes	53.1	5.34 (2.74–10.39)		3.47 (1.27–9.50)	

Block 2.3 – Variable related to the health of the older adult: geriatric syndromes					

Fall^a^			0.002		0.754
No	28.4	1		1	
Yes	45.5	2.10 (1.32–3.33)		1.15 (0.65–4.72)	
Functional capacity to carry out IADL^a^			< 0.001		0.050
Independent	23.0	1		1	
Dependent	91.2	34.72 (13.31–90.55)		5.84 (1.00–34.27)	

IADL: instrumental activities of daily living

^a^ p-value for heterogeneity.

^b^ p-value for linear trend.

## DISCUSSION

Prevalence of frailty was 35.7%, which is similar to that found in other national studies that have used the same tool to operationalize the outcome. In the study of adaptation and validation of the EFS in a sample of Brazilian older adults, prevalence was 31.4%^9^. In the research carried out by Fhon et al.^10^, 39.1% of the subjects presented some degree of frailty. Prevalence was 30.1% in another study carried out with older adults treated at a FHU unit in a municipality of São Paulo, Brazil^9^. Duarte et al.^7^ estimated a prevalence of 39.2% when investigating frailty in a sample of older women.

Discrepancies in prevalence, varying between 4.0% and 59.1%, are also found in the literature^3^ ^,^ ^4^ ^,^ ^11^ ^,^ ^12^ ^,^ ^24^. However, it is important to note that these divergences can be attributed to the different theoretical models and the operational tools adopted to measure frailty, as well as the issues of methodological nature, such as type of study, characteristics, and selection criteria^3^ ^,^ ^4^ ^,^ ^11^ ^,^ ^12^ ^,^ ^24^. Frailty can be conceived as an evolving concept, as there is no common definition among researchers. This fact implies different tools and types of evaluation^3^ ^,^ ^4^ ^,^ ^8^ ^,^ ^11^ ^,^ ^22^ ^,^ ^25^. Therefore, there is a wide range of possibilities to explore and interpret this syndromic condition, which makes direct comparisons between the prevalence estimated by the studies questionable. Researchers in the area also say that, given the lack of common definition surrounding the subject, it is important to keep in mind that the available evaluation tools are not exclusive but complementary^3^ ^,^ ^4^.

Other studies indicate an association between gait difficulty and use of auxiliary device and the evaluated outcome^12^ ^,^ ^24^. These factors are directly related to the physical dimension of frailty. The triad of sarcopenia, neuroendocrine dysfunction, and immune alteration has been proposed as a vicious cycle of gradual decline of energy, resulting in increased dependence and susceptibility to aggressors^11^. Sarcopenia is characterized by the progressive and generalized loss of skeletal muscle mass and muscle function (strength or performance) associated with aging; consequently, it generates weakness, fatigue, and reduced tolerance to exercise and ability to perform daily activities^5^. The reduction in neurocognitive speed is also indicated as an important component of frailty in older adults, capable of predicting slow gait^23^. In this way, frail individuals report difficulty in walking and need for an auxiliary device to walk. Environmental issues and frequent health conditions in older adults, such as falls, morbidities, functional dependence, and fear of falling can also explain this difficulty and the use of assistive technology.

Depressive disorders and depression are strongly related to frailty, falls, and decline in functional capacity and quality of life. Older adults with depressive disorders tend to reduce the level of physical activity, social participation, autonomy, and independence in carrying out activities of daily living, contributing to the perpetuation of the vicious cycle of frailty^6^ ^,^ ^11^ ^,^ ^14^ ^,^ ^24^. In addition, cognitive impairment is commonly found in older adults associated with different types of depressive disorders^6^. The literature also reports that, there is an increasing tendency for depressive symptoms to be developed as the level of frailty increases^11^ ^,^ ^15^ ^,^ ^24^.

We observed greater frailty among older adults who have caregivers in this study. With the increase in frailty among older adults and the intensification of this syndromic condition, we can expect a greater degree of functional dependence, greater use of health services, and greater demand for health care, social support, and institutionalization^3^ ^,^ ^4^ ^,^ ^21^ ^,27^. In our study, as reported in the literature, a family member or friend takes responsibility for caring for the older adult^18^. Keeping the older adult in the community, having a caregiver to supervise him or her, and providing the necessary health and social support can be an important alternative to avoid institutionalization and to maintain quality of life. The higher prevalence of frailty among those who have caregivers can also be explained by the fact that the need for caregiver is a consequence of frailty. However, we highlight that no studies were found that have specifically explored the relationships between frailty and caregiver.

This study corroborates the association between frailty and functional impairment widely discussed in the literature^10–12,14,17,21,24,26^. The frailty syndrome is considered a dynamic process, leading to a spiral of decline of several systems, responsible for increasing the status of frailty and for promoting or intensifying other conditions, such as autonomy and functional disability. Both functional disability and frailty can be understood as a complex construct, resulting from the interaction and the way in which various factors, whether biological, psychological, clinical, social, or environmental, are articulated for each older adult. Predictors of mortality among older adults are also important^3^ ^,^ ^4^ ^,^ ^11^ ^,^ ^17^ ^,^ ^25^. Research studies in different populations also describe the existence of a growing association between a higher degree of functional impairment and higher levels of frailty^10^ ^,^ ^11^ ^,^ ^17^ ^,^ ^24^.

The use of EFS also allowed us to stratify the levels of frailty and to identify those who are considered as apparently vulnerable, also called pre-frailty, who are at high risk of evolving to a status of frailty. The values estimated in this research corroborate the data available in the literature^7^ ^,^ ^8^ ^,^ ^10^. Although the apparently vulnerable group is the one most at risk of progressing to frailty, we need to consider that the course and the development of this syndrome are extremely variable and affected by the complex network in which the individual is placed. This condition is reversible, especially in the early stages, and even in more advanced stages, the reduction of the severity of the condition can also be achieved, allowing benefits for the older adult, family, society, and social and health systems^3^ ^,^ ^4^ ^,^ ^a^.

This research was conducted with high methodological rigor. We took the necessary precautions in the sampling process and similar results were pointed out in the literature^7^ ^,^ ^8^ ^,^ ^10^. Considering the use of sample weights, even if the sample were to be expanded, little variation would exist for the parameters estimated in this study.

The use of the EFS is still incipient in Brazil and the main limitations of this study are associated with the history of fragility in the adaptation of the EFS to the Brazilian context and the use of the clock test, even as an integral part of the EFS, in populations with low education level.

With the demographic and epidemiological changes, we need to understand the health dynamics of the older population and how this relationship occurs at the individual and social levels, as well as in relation to the health and social systems. The WHO^a^ advocates the realignment of health systems, focusing on Primary Health Care, identification of the special needs of frail older adults, and changes that allow the sustainability of these systems. It also warns about the importance of recognizing frailty as a public priority.

Based on this perspective and the assumptions present in the Brazilian Health Reform, we consider the EFS as a particularly useful instrument for the systematic tracking and management of frailty in community-dwelling older adults; therefore, the EFS would fit into the context of Primary Health Care. It is an instrument compatible with the reality of the Brazilian system and it brings central and structural issues for the SUS in its concept, such as expanded conception of health, interdisciplinarity, and multidimensional approach. These characteristics allow the EFS to contribute with the universality, equity, and integrality of the care of the older adult.

The recognition of the most vulnerable groups and the understanding of the factors associated with frailty, considering their multifactorial nature, are primordial tools for the elaboration and implementation of actions and strategies of health prevention, rehabilitation, and promotion. They can also be used to plan health care models, which address the main problems that affect the older population.

## Footnotes

a World Health Organization. World report on ageing and health. Geneva: WHO; 2015 [cited 2017 Apr 20]. Available from: http://apps.who.int/iris/bitstream/10665/186463/1/9789240694811_eng.pdf

b Minas Gerais. Secretaria de Estado de Saúde. Manual do Prontuário de Saúde da Família. Belo Horizonte: SES-MG; 2008.

c Ministério da Saúde (BR), Secretaria de Atenção à Saúde, Departamento de Atenção Básica. Envelhecimento e saúde da pessoa idosa. Brasília (DF); 2006 [cited 2017 Apr 20]. (Caderno de Atenção Básica, 19). Available from: http://dab.saude.gov.br/portaldab/biblioteca.php?conteudo=publicacoes/cab19
